# Promiscuous nitrilase from *Bacillus subtilis* for herbicide degradation and plant growth promotion

**DOI:** 10.1038/s41598-026-52818-8

**Published:** 2026-05-12

**Authors:** Archana Kumari, Chiranjit Ghosh, N. Kannan, S. Balaji

**Affiliations:** Manipal Institute of Technology, Manipal Academy of Higher Education, Manipal, Karnataka 576104 India

**Keywords:** Dichlobenil, *Bacillus subtilis*, Nitrilase, Biodegradation, Accession Number PX891009, PGPR, SDG 2,3,6,12,15, Biochemistry, Biotechnology, Environmental sciences, Microbiology, Plant sciences

## Abstract

The growing use of nitrile-containing herbicides in agriculture has raised concerns about the environment and human health. In this research, the ability of the bacterial isolate to produce a thermostable enzyme that can degrade nitrile-containing compounds was analysed. The promiscuous nature of the nitrile-degrading enzyme was analysed based on substrate specificity. The enzyme exhibited a notable degradation profile on the nitrile-containing dichlobenil (526.3 µmol/min.mL) and the highest activity towards the native substrate acrylonitrile (555.5 µmol/min.mL). Additionally, divalent metal ions, including Ca^2+^, Mg^2+^, and Fe^2+^, increased the activity of the enzyme but Cu^2+^, Co^2+^, Mn^2+^, Zn^2+^, and K^+^ decreased it. The enzymatic breakdown of dichlobenil into its corresponding carboxylic acids was determined by FTIR and GC-MS/MS. The 16 S rRNA gene was used to characterise the bacterial isolate, and sequence similarities verified that it was *Bacillus subtilis*. Subsequently, the sequence was deposited in GenBank with the accession number PX891009. *B. subtilis* possesses plant growth-promoting traits such as indoleacetic acid and gibberellic acid, ammonia production, phosphate solubilization, and produces hydrolytic enzymes that stimulate defence mechanisms. These results suggest that *B. subtilis* has the potential to degrade nitrile-containing herbicides to improve soil fertility. This work also contributes to the Sustainable Development Goals (SDGs), especially SDGs 2, 3, 6, 12, and 15, by supporting the biological degradation of herbicides to enhance soil quality.

## Introduction

Organic pesticides represent one of the largest groups of xenobiotic compounds introduced into the environment for agricultural weed, insects, and fungi. It accounts for a significant proportion of the global pesticide market. It is estimated that the global herbicide market share was 51.9% in 2019, with a total pesticide market value of $84.5 billion^1^. It has been reported that the global herbicide market value is $ 32.3 billion in 2023, which is expected to grow at a CAGR of 6.3% from 2024 to 2034 and reach $ 63 billion by the end of 2034^2^. Among all the Asian countries, India ranks second in pesticide consumption, with > 62,193 tons of active ingredients used in agriculture^3^. Their primary function is to suppress the growth of weeds^4^. Over recent decades, increasing frequencies of pesticide residues have been reported in both surface and groundwater systems across the world. When these herbicides are applied in agricultural fields, they can contaminate surface water due to runoff^5,6^. In addition to the parent compounds, persistent transformation products formed during partial degradation have emerged as critical environmental concerns due to their enhanced mobility and resistance to further biodegradation. It has the potential to leach into the soil horizons and pollute groundwater sources when exposed to heavy rainfall^7^. The aquatic organism absorbs these chemicals and passes them to different trophic levels of the food chain^8,9^, which may lead to detrimental effects on human health^10,11^.

The environmental fate of benzonitrile herbicides is strongly influenced by their chemical structure and physicochemical properties. While bromoynil and ioxynil possess hydroxyl groups that enhance photodegradation and water solubility, dichlobenil is resistant to photolysis, contributing to its persistence in soil^12,13^. Moreover, metabolites of dichlobenil formed during degradation have substantially lower sorption coefficients and higher mobility, increasing their susceptibility to leaching into the groundwater systems. These properties highlight the limitation of abiotic degradation processes of converting benzonitrile herbicides into corresponding metabolites, such as benzamide, which is persistent in the environment, emphasising the importance of biological degradation that transforms benzonitrile herbicides into less toxic products^14–18^.

Benzonitrile herbicides such as dichlobenil and bromoxynil are toxic to the environment due to the presence of functional groups such as cyanide and dihalogenated phenyl rings^12,13^. Dichlobenil is used as a pre-emergence herbicide, whereas bromoxynil is a widely used post-emergent herbicide^14^. These herbicides have been classified as toxicity class II and may be carcinogenic, according to the U.S. Environmental Protection Agency (EPA, 1998). Most of the agricultural sites of India were found to have dichlobenil and bromoxynil topsoil deposition of 10–50 µg/kg, and the leaching rate is 1–5 µg/day^15^. These herbicides exhibit endocrine-disrupting effects on aquatic species, and long-term exposure to humans may result in cellular dysfunction, such as tachypnea, tachycardia, metabolic acidosis, elevated CO_2_ generation, and hyperthermia^19,20^. This research focuses on nitrile-degrading bacteria. There are enough studies on Bacillus sp. as a rhizobacterium that promotes plant growth (PGPR). Nevertheless, the biodegradation of nitrile-containing herbicides is limited^21^.

## Materials and methods

### Chemicals and reagents

Acetonitrile, Benzonitrile, Acrylonitrile, Acrylamide, Bisacrylamide, Bromoxynil and Dichlobenil from Sigma-Aldrich (Bangalore, India), and all standard chemicals for media preparation were purchased from Hi Media (Bangalore, India). Unless otherwise specified, all chemicals and reagents were of analytical grade.

### Microorganism and culture conditions

The bacteria isolated from fermented foods of North-East India were inoculated into mineral salt media (MSM). The composition of MSM is (%): K_2_HPO_4_, 0.68; KH_2_PO_4_, 0.12; magnesium sulphate heptahydrate, 0.01; manganese (II) sulphate tetrahydrate, 0.01; calcium chloride, 0.01; ferrous sulphate, 0.01; and sodium molybdate, 0.0006 ^16^. The MSM media was supplemented with 1% of acrylonitrile. The pH of the medium was adjusted to 7.0 before autoclaving at 121 °C for 15 min. After inoculation, the flasks were incubated at 37 °C on an orbital shaker at 170 rpm for 3 days. Then the supernatant was harvested by centrifugation at 10,000 rpm for 10 min at 4 °C. The growth profile of the microorganism was constructed, and a subculture was prepared and stored at 4 °C for future experiments.

### Screening for ammonia production

The screening of nitrilase-producing bacteria was performed using the nesslerization method, which quantified the ammonia generated^17^. The assay was carried out by the addition of 300 µL supernatant (crude enzyme), 200 µL of 150 mM acrylonitrile (substrate), and 500 µL of 10 mM phosphate buffer (pH 7.4). The reaction mixture was incubated at 37 °C for 30 min. The blank was prepared in the same way without adding crude enzyme. The ammonia liberated in the reaction was detected by 50 µL of Nessler’s reagent^18^.

### Morphological and biochemical characterisation

The morphological analysis of an isolated bacterial strain was performed using Gram staining techniques. The stained isolates were observed by light microscopy (Euromax, Holland). The biochemical characterisation, such as indole production, methyl red, Voges-Proskauer, citrate utilisation, triple sugar iron, xylanase production and starch hydrolysis, was analysed. After that, the screening of the isolated strain was assessed using the standard procedures^22^.

### Identification and characterisation of bacterial isolate M7

The characterisation of bacterial isolate (M7) was performed through PCR amplification of the 16 S rRNA gene, utilising a 25 µL reaction mixture including 0.4 mM of each deoxynucleoside triphosphate (dNTPs), 3% Dimethyl sulfoxide, 10 µM forward primer 357 F (5-CCTACGGGAGGCAGCAG), and 10 µM reverse primer 780R (5-TACCAGGGTATCTAATC), 5 U Taq polymerase and 1 µL of template DNA. The amplification of DNA was done with Veriti thermocycler using the following cycle conditions: initial denaturation at 95 °C for 5 min followed by 30 cycles of denaturation at 95 °C (30 s), annealing at 60 °C (1 min), and extension at 72 °C (90 s). A final extension at 72 °C was set for 15 min. The amplified DNA products were analysed on a 1.5% agarose gel, performed at 100 V for 30 min.

### Sequence analysis and phylogeny

The sequence analysis was done using BLASTN with default parameters. The 16 S rDNA matching sequences such as *B. subtilis*, *B. spizizenii*, *Geobacillus*, *B. firmicutes*, and *Bacillus*, were retrieved by selecting the FASTA (aligned sequence) and downloaded as Multi-FASTA Format. This is considered an ingroup taxon. The *Lactiplantibacillus* strain is included as an outgroup for phylogenetic tree construction. The phylogenetic tree was constructed using Data Analysis in Molecular Biology and Evolution (DAMBE ver.7.3.32)^23,24^. The consensus tree is created based on the NJ-method with input order randomised. The sequences are bootstrapped 100 times, and the distance used was MLCompositeTN93^25,26^.

### Quantitative detection of nitrilase

Enzyme activity was evaluated by determining the production of ammonia following the modification of the phenol/hypochlorite method^27^. For the reagent, the following components were used. Reagent A contained 0.6 M phenol and 0.001 M sodium nitroprusside. Reagent B contained 0.11 M sodium hypochlorite and 2.1 M sodium hydroxide. The standard assay was done in triplicate at 37 °C in a reaction containing 180 µL potassium phosphate buffer (0.1 M, pH 7.5), 100 µL enzyme, and 100 µL of the 150 mM acrylonitrile. Samples were incubated at 37 °C for 30 min. The reaction was quenched by the addition of 100 µL of the assay mixture to 300 µL reagent B, followed by the rapid addition of 300 µL reagent A with vigorous mixing and incubation at room temperature for 15 min^28^. The intensity was estimated at 700 nm by using a spectrophotometer (Eppendorf). One unit of nitrile-degrading enzyme activity was defined as the amount of enzyme capable of releasing one µmol ammonia in one minute under standard reaction conditions^27^. The degradation of herbicides was also assessed using both qualitative and quantitative colorimetric methods.

### Optimal parameters of nitrile-degrading enzymes

Enzyme activity has been evaluated at 37 °C in buffers with pH values between 3 and 9 to determine the influence of pH on enzyme activity. The optimum temperature for the enzyme activity was determined by a standard enzyme assay conducted at temperatures of 30–80 °C. The enzyme activity was quantified as a percentage of relative activity in relation to the maximal activity achieved at a certain temperature^29^. The effect of metal ions on enzyme activity was assessed by pre-incubating the enzyme with mono and divalent metal ions (Na^+^, K^+^, Ca^2+^, Mg^2+^, Cu^2+^, Fe^2+^, Mn^2+^, Ni^2+^, and Co^2+^) and the control was done in the absence of metal ions.

### Enzyme inhibition

The presence of nitrilase activity was confirmed by the competitive inhibitor benzaldehyde. In the enzyme reaction mixture, 100 µL of bacterial supernatant was mixed with 180 µL of 50 mM phosphate buffer (pH 7.5), which included 100 µL of the 150 mM acrylonitrile as the substrate. The reaction lasted for an hour at 28 °C. Benzaldehyde, which is known to inhibit nitrilase^30^, was introduced at varying concentrations (0.1–5 mM), and the effect of the reaction was observed. In the control, the inhibitor was excluded. Following incubation, phenol/hypochlorite reagents were administered to the reactions. The intensity was measured at 700 nm using a spectrophotometer (Eppendorf, basic model). The inhibition of enzyme activity was assessed by a reduction in the yield of acrylic acid.

### Substrate specificity

For the determination of the substrate specificity, nitrile-containing substrates were chosen, such as Acetonitrile, Benzonitrile, Acrylonitrile, Acrylamide, and Bisacrylamide (100–1000 mM). Besides these substrates, Bromoxynil and Dichlobenil (herbicides) were also included as one of the substrates to analyse enzymatic degradation. Kinetic constants (Km and Vmax) were determined from a Lineweaver–Burk plot, based on substrate specificity and concentration.

### Analysis of pesticide degradation

The degraded dichlobenil was examined using Fourier transform infrared spectroscopy (FTIR) and gas chromatography- mass spectrometry (GC-MS)^31–33^. FTIR analysis was performed using a Shimadzu Fourier Transform – Infra-Red Spectrophotometer (IRSpirit-T) with a QATRTM-S Single-Reflection ATR assessor with a diamond crystal. The scan rate was established at 4 cm⁻¹ per second. The gas chromatography-mass spectrometer (Agilent GC8890-MS7000E) was employed to identify dichlobenil degradation in the sample. Mass spectrometric conditions used for GC-MS/MS analysis are shown in Table 1. The DB-5 column (Agilent, size: 30 m x 0.25$\:\mu\:$m x 0.25 $\:\mu\:$m) was used for the study. The injection temperature was set at 250°C was used in splitless injection mode was used. The flow rate of the helium stream was kept at 1.5mL/min. Elcometer 4340 was utilised to fabricate thin-film solid-phase extraction analytical patches, and the coated patches were used for thermal desorption of the analytes in GC-MS/MS. During sample preparation, a vortex mixer (REMI-CM-101 plus) and a shaking incubator (REMI-CIS-18plus) were used for extracting compounds onto the analytical patches.

**Table 1:** GC-MS/MS Equipment Operating parameters.

Sample	Liquide syringe automation (PAL-3, Agilent GC 8890 − 7000 EMS
Column	DB-5 capillary column (Agilent, size: 30 m x 0.25$\:\mu\:$m x 0.25$\:\mu\:$m)
Run time	30 min
Mass analyzer	Triple quadrupole tandem mass spectrometer
Mass range	30–300 AMU
Gass flow rate	1.5 mL/minute (Helium)
Injectore port temperature	250°C

### Plant growth-promoting traits

The *Bacillus* sp. qualitatively exhibited plant growth-promoting activities, including the production of indole acetic acid (IAA), gibberellic acid (GA), phosphate solubilization, and assays for catalase, cellulase, and protease.

#### Indole acetic acid production

The isolate was tested for IAA production by colourimetric assay^34^. The isolate was grown in 50 mL Luria-Bertani broth (pH 7.5) containing 0.1% (w/v) tryptophan and inoculated with 1% (v/v) inoculum and incubated at 37°C in the dark, at 170. After incubation, 1 mL of supernatant was mixed vigorously with 4 mL of Salkowski’s reagent (150 mL conc. H_2_SO_4_, 250 mL H_2_O, 7.5 mL 0.5 M FeCl_3_. 6H_2_O), incubated for 30 min and the absorbance was measured at 520 nm with the help of a spectrophotometer. The concentration of IAA produced by the bacterial isolates was determined from a standard curve generated using a standard solution of commercial IAA.

#### Gibberellic acid production

Culture media were filtered, and then samples were acidified to pH 2.5 with HCl and extracted using liquid-liquid (Ethyl acetate/NaHCO_3_) extraction. Gibberellic acid in the ethyl acetate phase was measured by a UV spectrophotometer at 254 nm. The amount of gibberellic acid was calculated from the standard curve^35^.

#### Phosphate solubilization

The potential of *B. subtilis* to degrade phosphate-containing substrates was analysed. The bacterial culture was inoculated in sodium phytate-containing MSM agar plate and incubated at 37 ℃ for 48 h. The formation of a clear zone around the bacterial colony indicates phosphate solubilization potential^36^.

#### Qualitative analysis of hydrolytic enzymes

The 1 mL of bacterial culture was treated with 2–3 drops of 3% H_2_O_2_ in a test tube. Effervescence demonstrated the release of oxygen, indicating catalase activity^37^. The cellulase activity of the bacteria was analysed. The MSM agar with 1% CMC was prepared, and 10 µL of inoculum was added to the centre of the plate. Incubated at 37 ℃ for 48 h. After the incubation time, the plates were covered with Congo red dye for 15 min, then drained and washed with 1 M NaCl. The clear zone surrounding the bacterial colony indicates the breakdown of cellulose^38^. The protease activity of the bacterial isolates was determined using the skim milk agar medium containing casein (0.5%),yeast (0.25%),skim milk (0.1%),glucose (0.1%),and agar (1.05%),incubated for 24–48 h to observe the clear zones^39^.

#### Seed germination test

Seeds of green gram (*Vigna radiata* L.) were collected from the local shop of Manipal, Karnataka. The seeds were washed thoroughly with distilled water. Then, the seeds were treated with 0.1% (v/v) of sodium hypochlorite solution for 2–3 min. Seeds were then cleaned with 70% (v/v) ethanol, followed by rinsing with sterile distilled water. Disinfected seeds were used for seed germination tests and pot experiments.

Seeds (50 numbers) were placed on sterile cotton in a petri plate. Pure bacterial culture was added to the petri plates containing seeds and incubated at room temperature. For comparative evaluation, untreated seeds and treated seeds with distilled water are used as controls. After 72 h, germinated seeds were counted, and the germination percent and seed vigour index were calculated by the following formula:

$$\:Germination\:percent=\frac{Seeds\:germination}{Total\:seeds}\times\:100\:\:$$

$$\:Seed\:vigor\:index=Seedling\:lenght\times\:Germination\:percentage$$

### Pot experiment

Four plastic disposable containers, measuring 6 cm in height and 15.5 cm in width, were utilised. The container was filled with sterilised soil (350 g each) and placed at ambient temperature on the balcony of the Biotechnology laboratory at Manipal Institute of Technology, Manipal. The treatment comprises (a) mung seed with distilled water, (b) with *B. subtilis*, (c) with herbicide, and (d) herbicide combined with *B. subtilis*. The plants were monitored daily, and after 16 days, they were harvested for the analysis of growth promotion parameters, including root length, shoot length, fresh weight, dry weight, leaf length, leaf width, leaf area, and chlorophyll content. The fresh weight was recorded immediately, and dry weight was measured by drying the plant samples in an oven at 60°C for 48 h until a consistent weight was attained^40^.

### Assessing the total chlorophyll content

Total chlorophyll content was assessed for the treated plants using the method described by Khan et al. Fresh leaves were crushed in 5 ml of 100% acetone and then filtered using Whatman paper to collect the filter extract in a separate test tube. The absorbance was checked at 645 nm and 663 nm wavelengths, respectively, by the spectrophotometer. Chlorophyll a, chlorophyll b, and total chlorophyll were determined using the following formula^41^.

$$\:Chlorophyll\:a=12.7\left(A663\right)-2.7\left(A645\right)$$

$$\:Chlorophyll\:b=22.9\left(A645\right)-4.7\left(A663\right)$$

$$\:Total\:chlorophyll=Chlorophyll\:a+Chlorophyll\:b$$

### Statistical analysis

Statistical analysis was performed in triplicate for each sample. All results were reported as mean ± standard error, and statistical evaluation was performed using one-way ANOVA in Microsoft Excel. The p-value was calculated to assess the significance of plant growth parameters. A p-value of < 0.05 was considered statistically significant.

## Result and discussion

### Microorganism and culture conditions

**Fig. 1:**
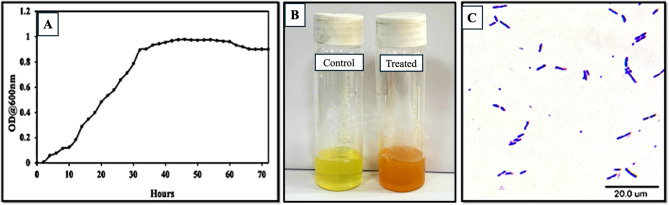
(**A**) Growth profile of *B. subtilis* in MSM with sole carbon source acrylonitrile. (**B**) Ammonia production is estimated by Nessler’s method. (**C**) Gram-positive rods.

The time course profile of the isolated bacterium (M7) is illustrated in Fig. 1(A), utilising acrylonitrile as the sole carbon source. The growth profile predicts a lag phase between 0 and 8 h, an exponential phase from 8 to 32 h, and an extended stationary phase from 32 to 70 h. This confirms that the isolate has utilised acrylonitrile. Further, the release of ammonia was qualitatively tested by observing the colour change, Fig. 1(B).

### Morphological, biochemical, and molecular characterisation

**Fig. 2:**
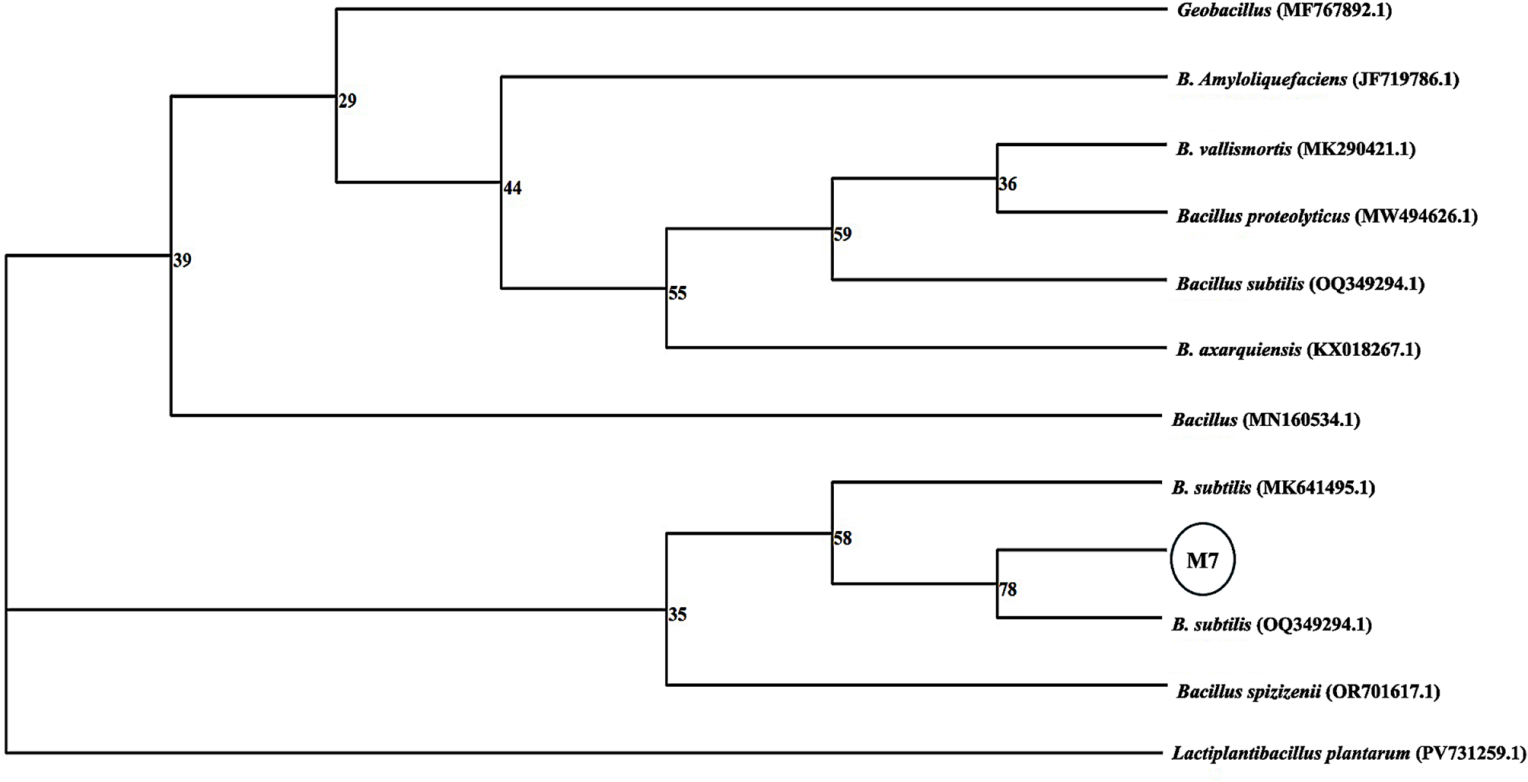
The phylogenetic tree was built using the FastME method with bootstrap (resampling 100 times). The relationship of M7 (Accession Number PX891009) with *B. subtilis* is shown. The values indicate the confidence interval on the nodes based on the bootstrap.

The morphology of bacterial isolate (M7) was identified to be Gram-positive and rod-shaped (Fig. 1C). The biochemical characterisation of isolate hydrolysed starch, gelatine, and casein. The isolate displayed positive responses for citrate, catalase, and urease tests. However, it is negative for the methyl red and indole tests. Based on the biochemical tests, the isolate probably belongs to the *Bacillus* genus. To confirm this, the sequencing of 16 S rDNA was done. The amplicon sequence (Accession Number PX891009) was queried against the core nucleotide BLAST database. The results were significant based on the E- value (0.0) and 99% query coverage. The top-ranked sequence with 99.52% identity with Accession Number OQ349294.1 confirms that our isolate is *Bacillus subtilis*. The phylogenetic position of the characterised isolate with other matching sequences is shown in Fig. 2.

### Enzyme activity

**Fig. 3:**
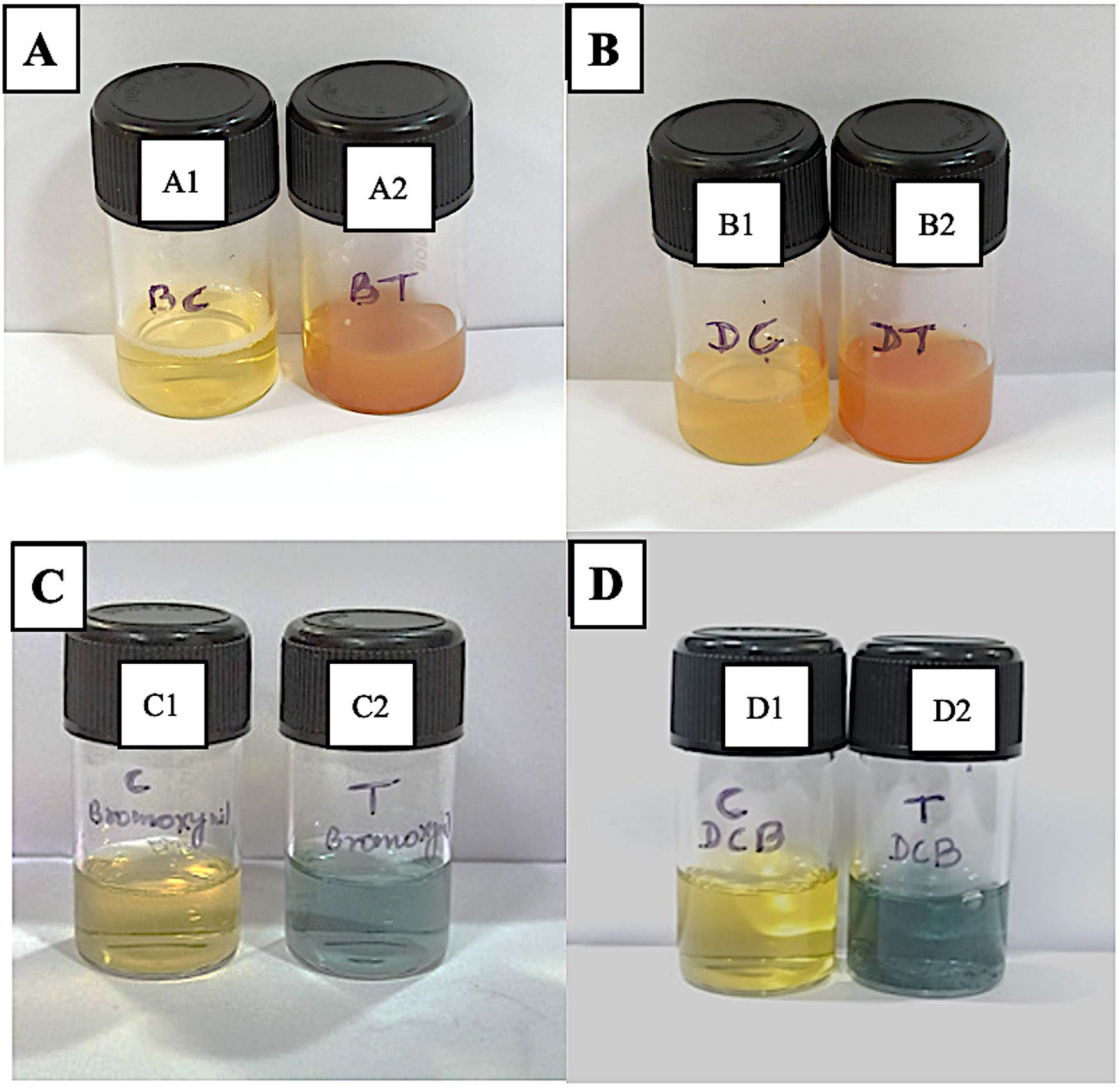
Nessler’s colourimetric assay for ammonia detection (**A**) and (**B**). A1: control containing bromoxynil without enzyme, A2: bromoxynil with enzyme. B1: dichlobenil without enzyme, B2: dichlobenil with enzyme. (**C**) and (**D**) phenol/hypochlorite assay for quantitative estimation C1; Bromoxynil without enzyme, C2; bromoxynil with enzyme, D1; dichlobenil without enzyme, D2; dichlobenil with enzyme.

**Fig. 4:**
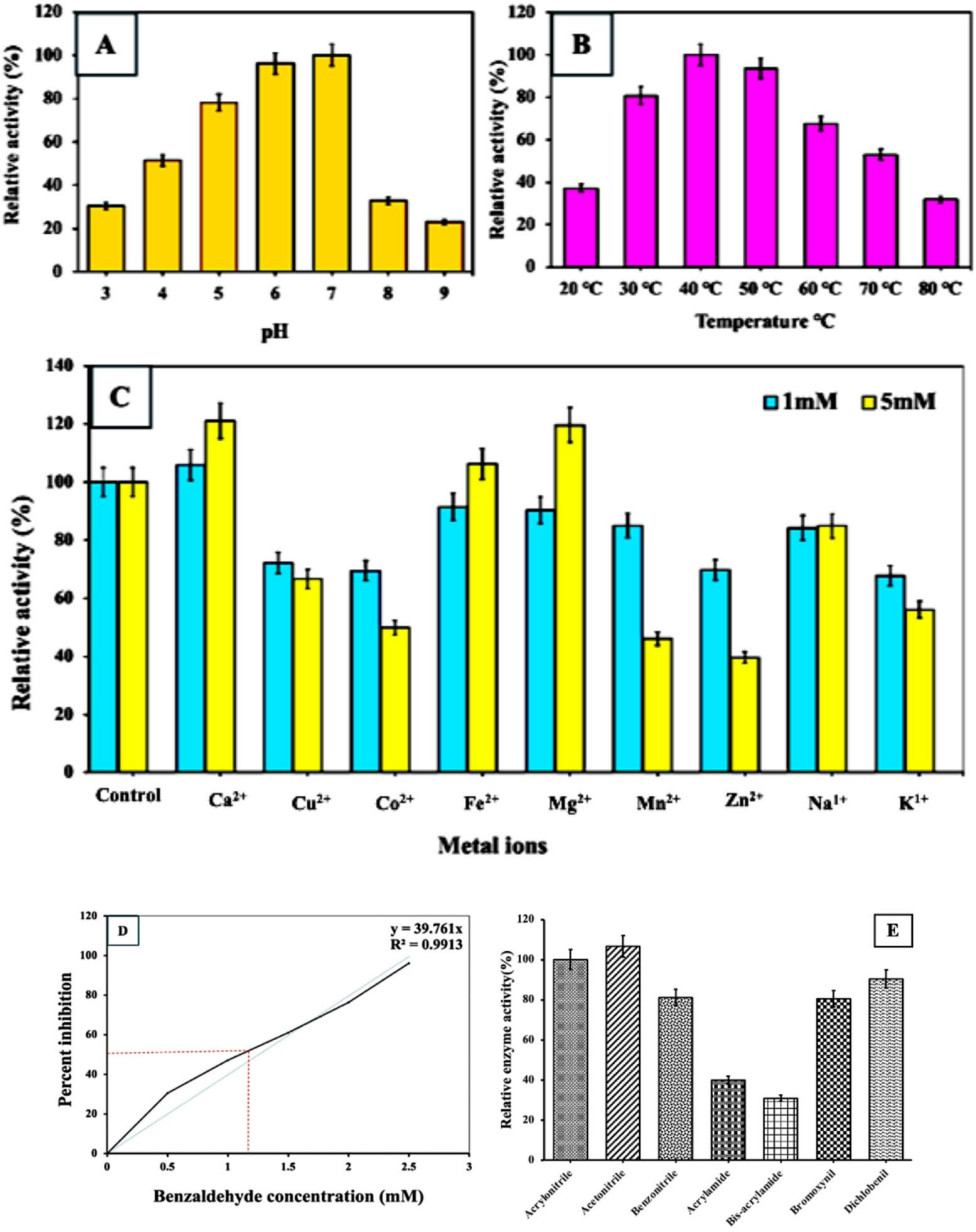
Effect of pH (**A**), temperature (**B**), and metal ion (**C**) on nitrilase activity. Benzaldehyde inhibition for nitrile-degrading enzyme (IC50 = 1.2 mM) (**D**). The broad substrate specificity of nitrile-degrading enzyme (**E**).

The *Bacillus subtilis* nitrile-degrading enzyme exhibited an activity of 289.9 $\:\mu\:$mol/ml/min using acrylonitrile as the substrate. The enzymatic activity of herbicides, including bromoxynil and dichlobenil, was measured at 269.14 $\:\mu\:$mol/ml/min and 322.35 $\:\mu\:$mol/ml/min, respectively (Fig. 3A-D). The enzyme demonstrated peak activity at pH 7, maintaining 78% of its maximum activity within the pH range of 5.0–7.0 (Fig. 4A). The activity diminished markedly below pH 5 and above pH 7. The thermostability was observed between 30 and 60 °C, showing a wide temperature range. The ideal temperature for the nitrile-degrading enzyme was 40 °C, and it maintained 98% activity at 50 °C, as illustrated in Fig. 4B. The nitrile-degrading enzyme exhibited positive and incremental activity with Mg²⁺ (19%), Fe²⁺ (6%) and Ca²⁺ (21%), while demonstrating inhibitory effects on Cu²⁺ (34%), K⁺ (44%), Co²⁺ (51%), Mn²⁺ (54%), and Zn²⁺ (61%), suggesting that metal ions can exert both activating and inhibitory influences based on their specific properties (Fig. 4C). The release of ammonia demonstrated indirect correlation with the increase in benzaldehyde concentration. The inhibitory concentration (IC_50_) of benzaldehyde was determined to be 1.2 mM (Fig. 4D). Previous studies have demonstrated^30^ that benzaldehyde effectively interferes with nitrilase activity. Furthermore, benzaldehyde significantly inhibits nitrilase activity^42,43^. This conforms with our results that the breakdown of acrylonitrile and other nitrile compounds in this investigation was facilitated by enzymatic hydrolysis.

### Substrate specificity

**Fig. 5:**
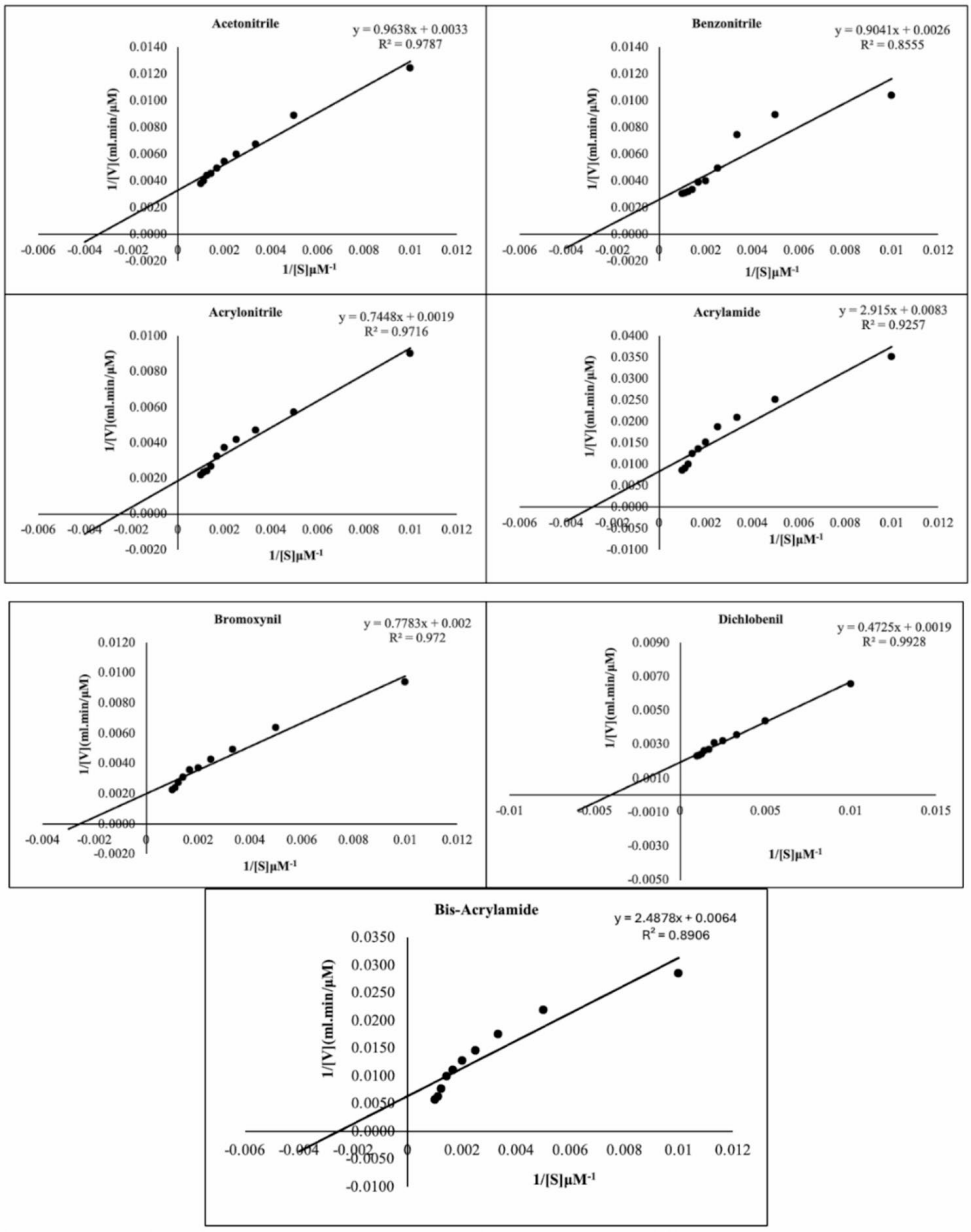
Lineweaver-Burk plot of the nitrile-degrading enzyme for different substrates.

The broad substrate specificity of the nitrile-degrading enzyme of *B. subtilis* was assessed with various nitrile and amide-containing substrates (Fig. 4E). The substrate specificity was classified based on the degradation efficiency and arranged in the following order: acetonitrile > acrylonitrile > dichlobenil > bromoxynil > benzonitrile > acrylamide > bis-acrylamide. The catalytic activity and kinetic parameters (K_m_ and V_max_ ) for the various substrates were evaluated using the Lineweaver–Burk plot (Fig. 5). The affinity towards the various substrates was identified by Km of 248.68 µM (dichlobenil) to 415.5 µM (acrylonitrile), and interpreted that dichlobenil had maximum affinity among all substrates. However, the Vmax (526.32 µmol/min.mL) shows high catalytic efficiency. Conversely, the acrylonitrile and bromoxynil showed relatively higher Km values, indicating lower binding affinity. However, the highest Vmax was observed for acrylonitrile (555.5 µmol/min.mL), followed by dichlobenil (526.3 µmol/min.mL) and bromoxynil (500 µmol/min.mL). This may pave the way for bioremediation.

### FTIR analysis of herbicide degradation

**Fig. 6:**
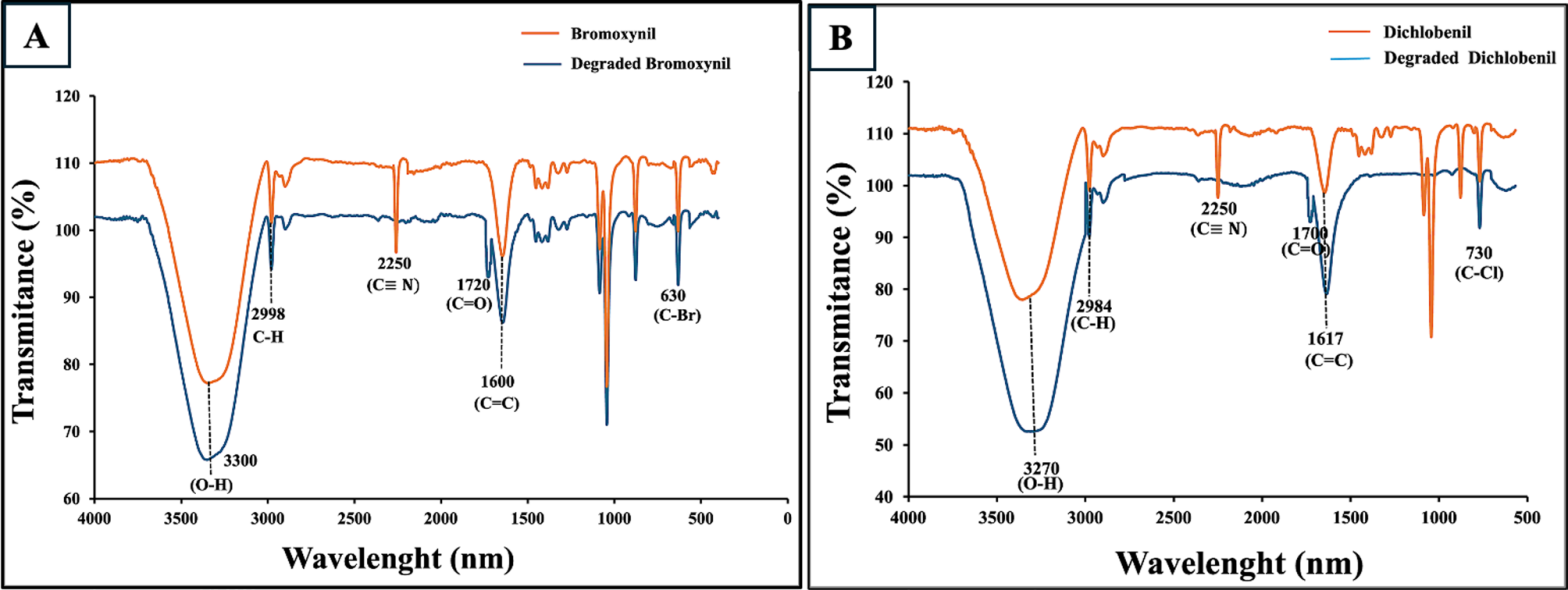
The FTIR spectrum of herbicide degradation. (**A**) Bromoxynil before degradation and after degradation, (**B**) dichlobenil before degradation and after degradation.

The FTIR spectra of bromoxynil and the enzymatically degraded product are shown in Fig. 6. The strong absorption peak at 2250 cm⁻¹ corresponding to the nitrile group (C ≡ N) of bromoxynil disappeared in the spectrum of the degradation product, dibromo-benzoic acid, indicating successful hydrolysis of the nitrile group. Additionally, the appearance of peaks at 3300 cm⁻¹ and 1720 cm⁻¹ confirms the presence of hydroxyl (O–H) and carbonyl (C = O) groups of carboxylic acid, respectively. The C–Br stretching appeared at 630 cm⁻¹ and aromatic C = C at 1600 cm⁻¹ in both spectra. For 2,6-dichlorobenzonitrile, the nitrile peak at 2250 cm⁻¹ disappeared after degradation, and the degradation product, 2,6-dichlorobenzoic acid, showed prominent peaks at 3270 cm⁻¹ (O–H) and 1700 cm⁻¹ (C = O), indicating formation of a benzoic acid derivative. The C–Cl stretch remained at 730 cm⁻¹, and aromatic ring vibrations were observed near 1617 cm⁻¹ in both compounds.

### GC-MS/MS analysis of herbicide degradation

**Fig. 7:**
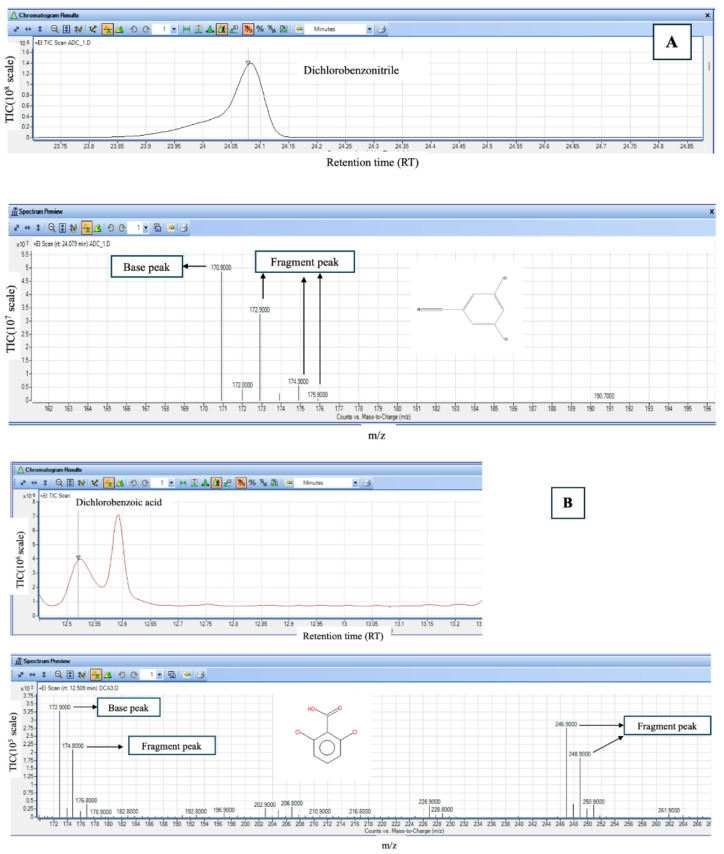
The GC-MS profile shows the degradation products of dichlorobenzonitrile. (**A**) dichlorobenzonitrile before enzyme degradation, (**B**) dichlorobenzoic acid product after enzyme degradation.

In this study, dichlobenil was selected due to its environmental persistence and its tendency to form water-soluble metabolites, which pose long-term risks to soil and water quality. The identification of degraded products of dichlorobenzonitrile through GC-MS/MS analysis is shown in Fig. 7. The compound dichlorobenzonitrile (CAS No. 6575-00-4) was identified at RT = 24.08 min with a reported base peak of 171. In the case of the enzyme-treated sample, dichlorobenzoic acid (CAS no 113882-34-1) was observed as a degraded product from dichlorobenzonitrile at RT 12.52 min (base peak 173). The metabolites were identified and validated by comparing their molecular weights, base peaks, and fragment ion peaks with the NIST library database (https://webbook.nist.gov/chemistry/). Similarly, it has the potential to degrade the bromoxynil. As the preliminary study showed, the production of ammonia. In substrate specificity, enzyme kinetics, and FTIR, the degradation of bromoxynil has been observed.

### Plant growth-promoting traits

**Fig. 8:**
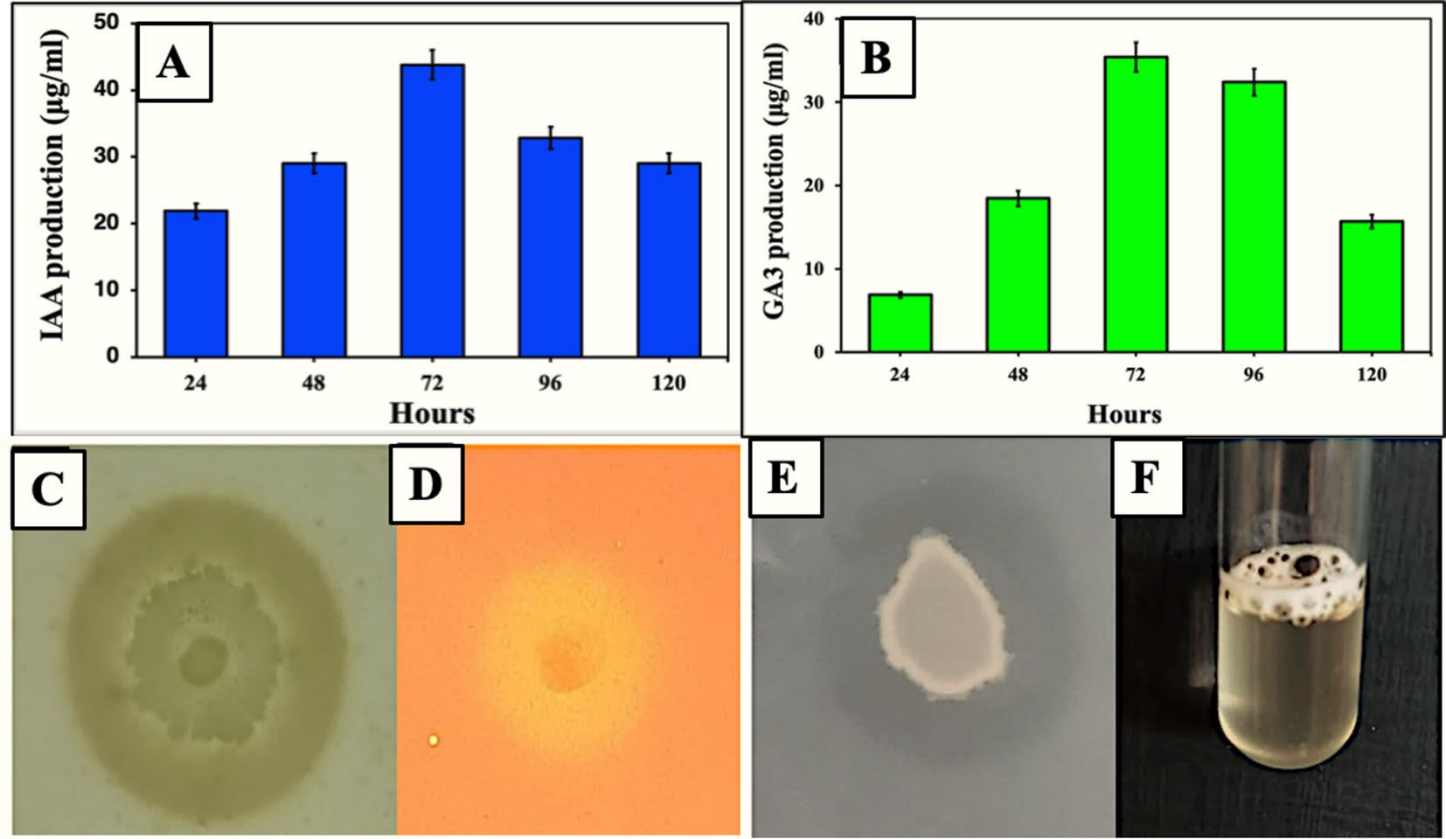
(**A**) IAA production from *B. subtilis*, (**B**) GA_3_ production from *B. subtilis*. (**C**) zone of clearance of phosphate solubilization, (**D**) protease, (**E**) cellulase, and (**F**) catalase.

The isolated *B. subtilis* possesses different plant growth-promoting traits such as IAA, GA_3_, ammonia production, phosphate solubilization, and produces different enzymes that stimulate the defence mechanism. The *B. subtilis* produced 44 $\:{\upmu\:}\mathrm{g}$/mL IAA and 35$\:{\upmu\:}\mathrm{g}$/mL GA_3_ after 72 h of incubation (Fig. 8A, B). The *B. subtilis* exhibited protease, cellulase and phosphatase activity (Fig. 8C-E). The catalase activity was validated by the effervescence produced by *B. subtilis* and its capacity to liberate oxygen (Fig. 8F).

### Seed germination

**Fig. 9:**
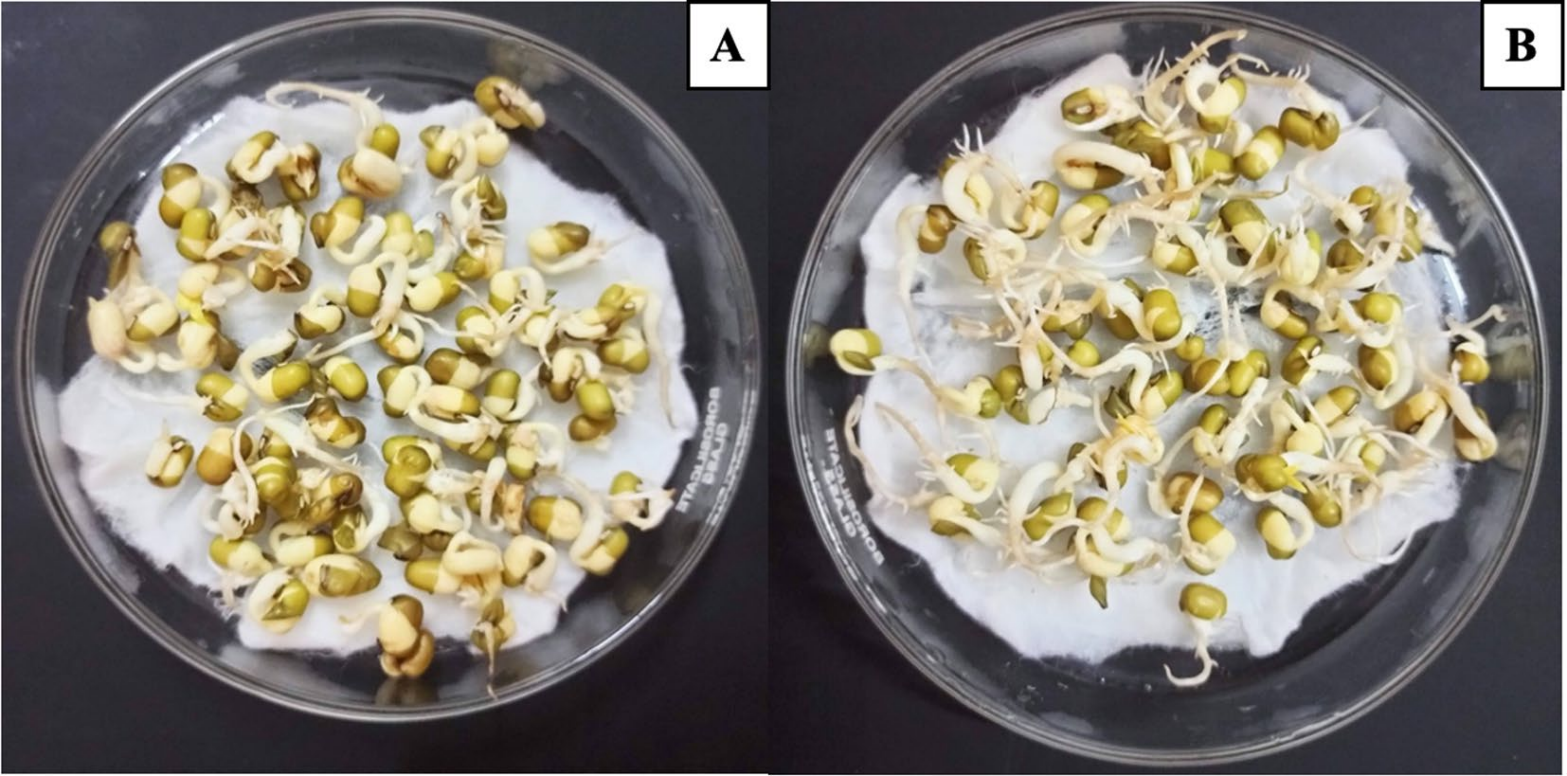
Seed germinated, (**A**) control, (**B**) seeds treated with *Bacillus subtilis*.

Seed germination in the presence and absence of *B. subtilis* is shown in Fig. 9. *B. subtilis* has a beneficial effect on mung seed germination, and it may be supplemented to promote plant growth in the field. The mung seeds treated with *B. subtilis* showed 95% seed germination with a seed vigour index of 154. In contrast, the control showed 75% and 110, respectively. Seed germination is the primary significant parameter to enhance and improve the total biomass and yield^44^.

**Fig. 10:**
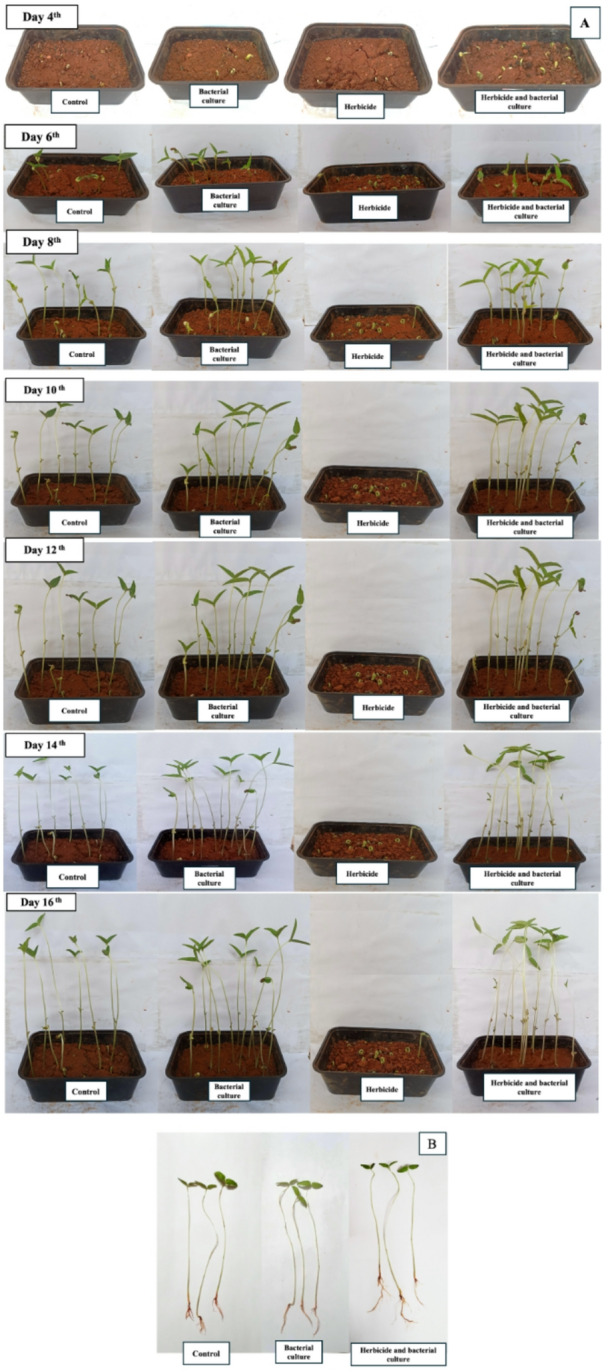
Sequential images showing the growth of green gram (*Vigna radiata* L.) seedlings taken every alternate day from day 0 to day 16 under natural conditions. **A**.) Four treatments were given: (1) water, (2) Bacterial culture, (3) Herbicide, and (4) Herbicide with bacterial culture. **B**.) Visual comparison of seedling growth under different treatments.

The evaluation of plant physiological parameters across different treatments such as water, bacterial culture, herbicide (dichlobenil), and herbicide with bacterial culture revealed significant differences in growth and development (Fig. 10). Plants treated with herbicide were showing reduced growth whereas the plant treated with herbicide along bacterial culture exhibited the highest shoot height (33.37 cm) and root height (10.30 cm), indicating enhancement in vegetative growth compared to the control (22.9 cm shoot, 4.47 cm root) and bacterial culture alone (26.7 cm shoot, 6.27 cm root). Moreover, fresh and dry biomass were highest in the herbicide with bacterial culture group (0.31 g and 0.20 g, respectively), compared to bacterial culture alone (0.24 g and 0.09 g) and the control (0.14 g and 0.08 g). Leaf morphological traits such as leaf length, width, and area were also significantly improved in the herbicide with bacterial culture treatment (3.07 cm, 1.4 cm, and 5.67 cm², respectively), compared to water (2.7 cm, 1.133 cm, 2.67 cm²) and bacteria alone (2.90 cm, 1.127 cm, 4.33 cm²). The total chlorophyll content was highest in plants treated with bacterial culture alone (7.35 mg/mL), followed by herbicide with bacterial culture (6.28 mg/mL), and lowest in the control (5.43 mg/mL).

**Table 2:** The results of ANOVA performed across the three treatment water, bacterial culture and bacterial culture with herbicide.

Source of Variation	SS	df	MS	F	*P*-value	F crit
Rows	24.69872	2	12.34936	3.541065	0.0533	3.633*
Columns	1767.337	8	220.9172	63.34594	0.0000*	2.591*
Error	55.79955	16	3.487472			
Total	1847.836	26				

The model and parameters were found to be statistically significant, except that the leaf number is persistently constant across all the treatment groups (Table 2). There are four parameters: shoot height, root height, fresh weight, and total chlorophyll content, which were highly significant (* p < 0.00*) and leaf length, width, and area demonstrated a trend toward significance.

Out of the treated group, bacterial culture and bacterial culture with herbicide treatments had significant improvement in growth compared to the untreated plants. The enhancement is due to the utilisation of herbicide by the microorganism and initiates the reduction of the nitrile group present in the herbicide, which in turn improves the availability of nitrogenous composition for the growth of the plant.

**Fig. 11:**
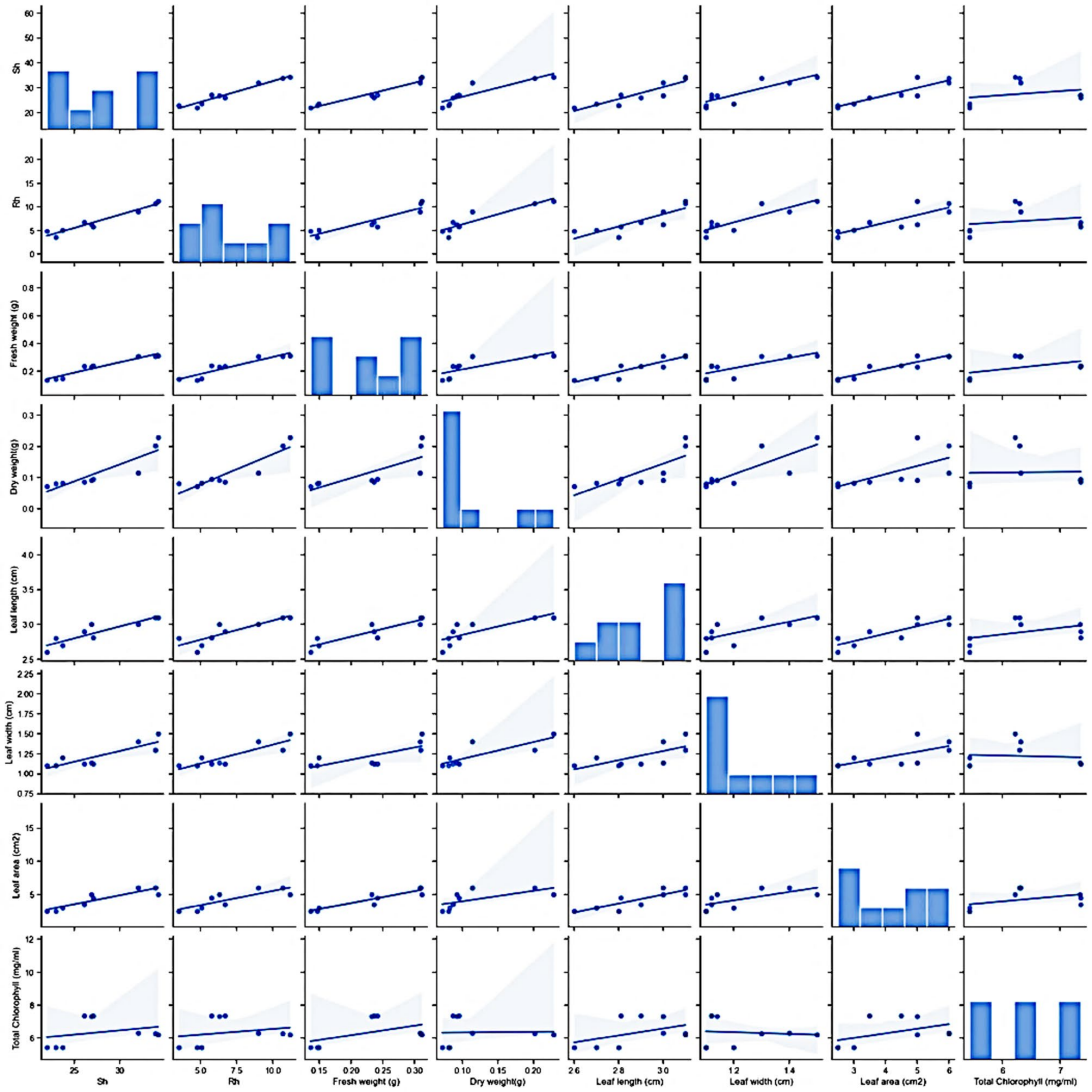
Correlation matrix of plant growth and physiological parameters under different treatments with bacterial culture and herbicides. The “scatterplot matrix” are plotted using Seaborn (a Python visualization library based on matplotlib) to show pairwise plots (using PairGrid option).

The correlation among the *Vigna radiata L.* growth parameters in plants treated in different conditions is showing positive relationship **(**Fig. 11**)**. The shoot height showed positive correlation with fresh weight (R² = 0.98), leaf width (0.99), leaf length (0.98), and total chlorophyll content (0.98), suggesting that taller plants had wider leaves, more biomass, and higher chlorophyll content^45,46^. Similarly, Root height exhibited correlations with dry weight (0.96), fresh weight (0.98), and leaf area (0.96). The fresh weight correlated with most traits, particularly leaf area (0.98) and shoot height; dry weight showed a lower correlation compared to fresh weight and leaf width. Similar relationships between biomass and growth parameters have been reported in previous studies^47^. Total chlorophyll content was positively correlated with all other traits, especially leaf length (0.98), leaf width (0.96), and shoot height (0.98). This indicates that more vegetative growth also supports higher photosynthetic pigment accumulation. This observation is in agreement with earlier findings showing a positive correlation between chlorophyll content and plant growth traits under stress conditions^48^.

## Discussion

The *B. subtilis* acclimatised to the modified media containing acrylonitrile as a sole carbon source, indicating the potential to grow by degrading nitrile-containing compounds. This is consistent with a previous study on the nitrile-degrading efficiency of bacteria cultured in acetonitrile as a carbon source^49^. The substrate degradation was prominent up to 30 h of incubation in laboratory conditions. This may likely be due to substrate depletion over prolonged incubation. The inhibition of nitrilase was confirmed using benzaldehyde as an inhibitor. Nitrilase was competitively inhibited, and the observed inhibition pattern is consistent with earlier reports^43,50^. As previously suggested^30^, aldehydes react with active site cysteines to form thiohemiacetal, which mimics the tetrahedral intermediates in nitrilase catalysis^51^.

The extracellular nitrilase from *B. subtilis* showed broad pH and temperature tolerance (Fig. 4A&B). A similar trend was observed by *Alcaligenes faecalis*^52^, *B. cereus*^53–55^, *B. pallidus*^56^, *Pseudomonas aeruginosa*, *Pseudomonas putida*^27,57^ showing optimal nitrilase activity at neutral pH. These findings collectively support that bacterial nitrilase exhibits optimal activity under neutral pH conditions. The nitrilase activity observed at acidic to neutral pH is particularly advantageous for cultivated soils^58^, as soil pH fluctuations are minimal in open field environments^59^. Under these conditions, the enzyme efficiently degrades nitrile-containing herbicides in the rhizosphere. The thermal tolerance of nitrilase from *B. subtilis* is particularly relevant for agricultural applications, as it is closely related to soil temperature, especially in tropical and subtropical regions^60,61^. This ensures effective herbicide degradation under laboratory conditions.

The observed catalytic efficiency of nitrilase towards multiple nitrile-containing substrates highlights its broad substrate specificity, indicating substrate promiscuity. Among the substrates, the catalytic efficiency was higher for acrylonitrile, followed by dichlobenil and bromoxynil (Fig. 5), suggesting a preferential affinity towards simpler aliphatic nitriles compared to more structurally complex aromatic herbicides. This trend is consistent with earlier reports where nitrilase showed higher activity towards low molecular weight nitriles due to reduced steric hindrance and improved accessibility to active sites^62,63^.

The enzymatic degradation of bromoxynil and dichlobenil was further analysed by FTIR, which showed the disappearance of the characteristic nitrile (C ≡ N) stretching peak, along with the appearance of O–H and C = O stretching peaks, confirming the formation of carboxylic acids. These spectral changes indicate the hydrolysis of nitrile groups into corresponding acids; this result aligns with earlier reports^51,60^. Furthermore, the FTIR findings were strongly supported by GC-MS/MS analysis (Fig. 7), which confirmed the formation of 2,6-dichlorobenzoic acid as a major degradation product of dichlobenil. This identification of the product not only validates the degradation but also confirms the functional activity of the enzyme in transforming toxic herbicides into less harmful compounds. Similar degradation products have been reported in previous studies involving nitrilase-mediated transformation of dichlobenil^33,64,65^.

The role of metal ions such as Ca²⁺ and Mg²⁺ is to enhance microbial enzyme activity, which has been documented, particularly in agricultural systems where they contribute to improved enzymatic stability and soil quality^66^. In agreement with these observations, the present study demonstrated a significant enhancement of nitrilase activity in the presence of Ca²⁺, Fe²⁺, and Mg²⁺, suggesting that these metal ions may act as cofactors and facilitate optimal enzyme conformation and catalytic efficiency, as supported by previous reports^33,65^. Additionally, nitrile and cyanide-containing compounds can persist in soil through stable metal-cyanide complexes; the presence of nitrilase-producing bacteria may offer an advantage in mitigating nitrile toxicity^67^. In contrast, the metal ions such as Co²⁺, Zn²⁺and Mn²⁺ significantly inhibit nitrilase activity^53^. This inhibitory effect is consistent with previous findings reported for *B. cereus*^49^, indicating that certain metal ions can negatively regulate enzyme activity depending on their concentration.

Beyond its herbicide degradation capability, *B. subtilis* produced higher amounts of IAA and GA_3_ compared to previously reported *Bacillus* species^68,69^. The elevated production of these phytohormones within a short incubation period suggests a strong potential to promote root elongation and shoot development. In addition, the formation of a clear hydrolytic zone around *B. subtilis* showed its potential as a phosphate-solubilising bacterium, which is a critical factor for phosphorus bioavailability and soil mineralisation^70,71^. In addition, *B. subtilis* has the potential to produce catalase, cellulase, and xylanase that are involved in plant growth promotion (Fig. 8D-F). Based on these, *B. subtilis* exhibits plant growth-promoting traits that can be applied to the agricultural field. These findings are consistent with previous reports on *Bacillus spp.* including *B. subtilis*, *B. japonicum*, and *P. putida*, in enhancing growth and stress^72–74^.

Although the chlorophyll content is highest in plants treated with *B. subtilis* alone^75^, and similar studies on the effect of PGPR and chlorophyll content of legume crops such as mung bean, pea, and soybean for enhanced physiological functions were reported^76–78^. In contrast, a moderate reduction was observed when the plant was exposed to both herbicide and *B. subtilis.* The reduction may be attributed to the degradation of the nitrile-containing herbicide by bacterial nitrilase, which leads to the production of ammonia. It has been reported that excess ammonia in plant tissues has been shown to inhibit chlorophyll synthesis and promote chlorophyll degradation^79^.

**Fig. 12:**
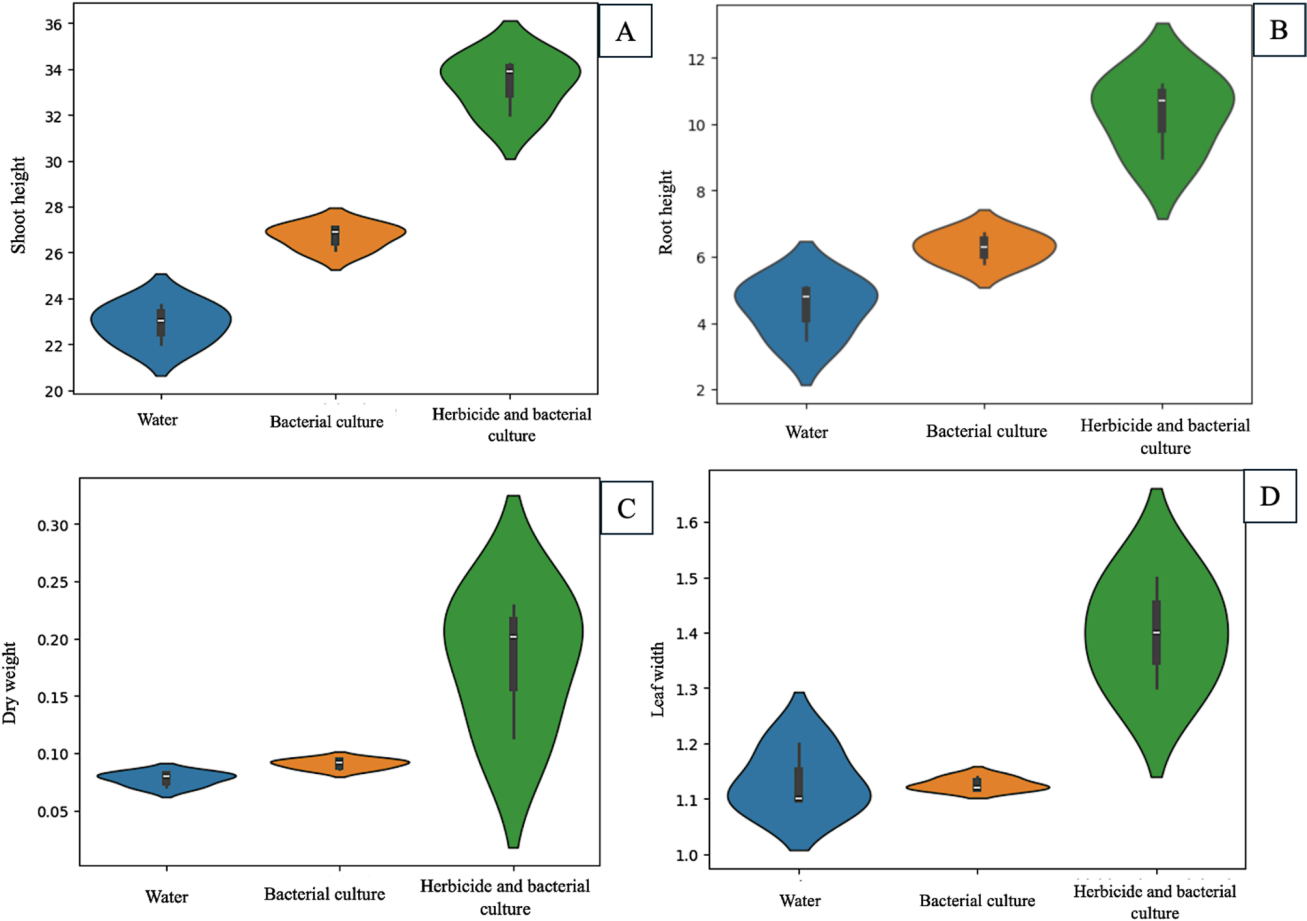
Violin plot showing (**A**) the distribution of shoot length, (**B**) root length, (**C**) dry weight, and (**D**) leaf width (the viewer can imagine this plot analogous to a leaf).

Plant growth parameters, including shoot height (Fig. 12A), root height (Fig. 12B), dry weight (Fig. 12C), and leaf width (Fig. 12D), showed treatment-dependent variation. Plants grown under water exhibited lower shoot and root length, along with lower dry weight. Inoculation with bacterial culture alone significantly enhanced both root and shoot height, accompanied by a moderate increase in dry weight. The significant improvement in all parameters was observed under the combination of herbicides and bacterial culture. The increase in shoot height and root height in the presence of *B. subtilis* is consistent with the plant growth-promoting activity of *Bacillus* species, which enhances shoot and root elongation through the production of phytohormones such as IAA and GA_3_^80,81^. Herbicide exposure often suppresses shoot and root growth by disrupting cellular metabolism and oxidative balance^82^. However, the presence of nitrilase-producing *B. subtilis* likely reduced the phytotoxic effect, thereby lowering stress on root tissues. In parallel, bacterial catalase and other antioxidant enzymes may have contributed to the reduction of reactive oxygen species generated under herbicide stress, enabling sustained root growth^83,84^.

Similarly, the increase in biomass observed in the presence of *B. subtilis* is consistent with the well-documented role of PGPR in improving plant growth through phytohormone production^80,85^, which stimulates cell elongation, division, and overall plant vigour^81^. The substantially higher dry weight observed under herbicide and bacterial culture suggests that *B. subtilis* mitigates herbicide-induced growth inhibition. Herbicides are known to reduce plant biomass by disrupting metabolic pathways and inducing oxidative^82^. The nitrilase activity of *B. subtilis* is likely attributed to herbicide degradation, thereby reducing phytotoxic effects and enabling sustained biomass accumulation. Additionally, bacterial catalase activity may have lowered reactive oxygen species levels, preserving photosynthetic efficiency and carbon allocation to biomass^83,86^. Enhanced dry weight accumulation under a combination of herbicide and bacterial culture has important agronomic implications, as higher biomass is associated with improved plant growth and resilience under chemical stress. Similar increases in plant dry weight following PGPR inoculation under herbicide stress conditions have been reported in legumes, cereals, and horticulture crops^75,87^. Although the present observations are derived from short-term experiments, they provide mechanistic evidence supporting the role of *B. subtilis* in promoting biomass while alleviating herbicide stress.

Furthermore, the plants treated with water exhibit moderate leaf width, while in the presence of bacterial culture, the leaf width slightly increased. In contrast, plants exposed to the combination of herbicide and bacterial culture showed a marked increase in leaf width. The bacterial culture may mitigate the phytotoxic effect of the herbicide, thereby supporting improved leaf growth. This indicates a positive synergistic effect of *B. subtilis* in mitigating the herbicide residual contamination and promoting vegetative growth. These observations are consistent with previous studies demonstrating the growth-promoting potential of PGPR, including *Bacillus*^73^ and *Bradyrhizobium japonicum*^88^. Overall, the findings of this study highlight the role of *B. subtilis* as a potent PGPR capable of promoting plant growth even under stress conditions such as herbicide exposure. Furthermore, bioremediation employing live microorganisms for pollutant removal is typically associated with low costs when compared with existing large-scale treatment methods^89–91^.

## Conclusions

The present study provides an insight into nitrile-degrading bacteria that display PGPR characteristics. Based on the biochemical and molecular characterisation, the isolate is identified as *B. subtilis.* The nature of the enzyme was verified using benzaldehyde, a known competitive inhibitor of nitrilase, at different doses (0.1–5 mM). The substrate specificity analysis revealed the highest Vmax for acrylonitrile, followed by dichlobenil and bromoxynil, demonstrating the effectiveness of the enzyme in degrading various nitrile-containing substrates, including herbicides. The degradation was confirmed by the FTIR and GC-MS/MS. In addition to its biodegradation potential, *B. subtilis* also demonstrated characteristics that encourage plant growth, including increased plant height and leaf development. Therefore, *B. subtilis* plays a dual role in both promoting vegetative growth and mitigating toxicity of herbicides. These results imply that *B. subtilis* is a promising biocatalyst for sustainable herbicide degradation and agricultural bioaugmentation. This study has some limitations; it did not involve a detailed soil characterisation or residue profiling. More field-level research with varying environmental conditions will follow the pilot studies. Future research will try to apply bacteria on pesticide contaminated soil in agricultural fields to scale up the technique for practical application.

## Data Availability

The 16s rRNA sequence has been successfully deposited in the NCBI GenBank database and is publicly available under the accession number PX891009. The sequence can be accessed at https://www.ncbi.nlm.nih.gov/nuccore/PX891009.
